# A crossover pilot study evaluating the functional outcomes of two different types of robotic movement training in chronic stroke survivors using the arm exoskeleton BONES

**DOI:** 10.1186/1743-0003-10-112

**Published:** 2013-12-19

**Authors:** Marie-Hélène Milot, Steven J Spencer, Vicky Chan, James P Allington, Julius Klein, Cathy Chou, James E Bobrow, Steven C Cramer, David J Reinkensmeyer

**Affiliations:** 1 Université de Sherbrooke, Faculté de médecine et des sciences de la santé, École de réadaptation, Centre de recherche sur le vieillissement, 1036 Belvédère sud, Sherbrooke (Québec) J1H 4C4, Canada; 2 Department of Mechanical and Aerospace Engineering, University of California; Irvine, 4200 Engineering Gateway, University of California, Irvine, Irvine, CA 92697, USA; 3 Departments of Neurology and Anatomy & Neurobiology, University of California, Irvine, 843 Health Sciences Road, Hewitt Hall room 1331, Irvine, CA 92697, USA

**Keywords:** Robot, Training, Stroke, Function, Activity of daily living

## Abstract

**Background:**

To date, the limited degrees of freedom (DOF) of most robotic training devices hinders them from providing functional training following stroke. We developed a 6-DOF exoskeleton (“BONES”) that allows movement of the upper limb to assist in rehabilitation. The objectives of this pilot study were to evaluate the impact of training with BONES on function of the affected upper limb, and to assess whether multijoint functional robotic training would translate into greater gains in arm function than single joint robotic training also conducted with BONES.

**Methods:**

Twenty subjects with mild to moderate chronic stroke participated in this crossover study. Each subject experienced multijoint functional training and single joint training three sessions per week, for four weeks, with the order of presentation randomized. The primary outcome measure was the change in Box and Block Test (BBT). The secondary outcome measures were the changes in Fugl-Meyer Arm Motor Scale (FMA), Wolf Motor Function Test (WMFT), Motor Activity Log (MAL), and quantitative measures of strength and speed of reaching. These measures were assessed at baseline, after each training period, and at a 3-month follow-up evaluation session.

**Results:**

Training with the robotic exoskeleton resulted in significant improvements in the BBT, FMA, WMFT, MAL, shoulder and elbow strength, and reaching speed (p < 0.05); these improvements were sustained at the 3 month follow-up. When comparing the effect of type of training on the gains obtained, no significant difference was noted between multijoint functional and single joint robotic training programs. However, for the BBT, WMFT and MAL, inequality of carryover effects were noted; subsequent analysis on the change in score between the baseline and first period of training again revealed no difference in the gains obtained between the types of training.

**Conclusions:**

Training with the 6 DOF arm exoskeleton improved motor function after chronic stroke, challenging the idea that robotic therapy is only useful for impairment reduction. The pilot results presented here also suggest that multijoint functional robotic training is not decisively superior to single joint robotic training. This challenges the idea that functionally-oriented games during training is a key element for improving behavioral outcomes.

**Trial registration:**

NCT01050231.

## Introduction

Each year, about 795 000 Americans suffer from a stroke. Up to 70% regain some function, but 30% remain permanently disabled, making stroke one of the leading causes of serious, long-term disability [1]. Developing ways to promote faster and greater recovery following a stroke is a key focus in research and clinical settings. There is a general consensus that recovery in chronic stroke is increased when exercises provide high-intensity, repetitive practice of desired movements [2,3], and take a functional focus [4,5]. However, lack of time and resources due to cost constraints on health care reimbursement hinder therapists from providing such training. Therapeutic adjuncts, such as robotic devices, might help address this challenge [6].

While robotic devices can help provide intense, repetitive training, research comparing the impact of robotic devices and conventional therapy on behavioral improvement following stroke have yielded modest but positive results in terms of impairment reduction, but not functional improvement [2,7-12]. For example, in their study comparing the use of the MIT-MANUS to intensive and conventional therapist-directed training, Lo et al. found significant motor impairment reduction of the trained limb after 36 weeks of robotic training when compared to conventional therapy, corroborating the results of previous studies of robotic manipulators [9]. However, the impact of robotic manipulators on improvement of activities of daily living (ADL) following stroke is less clear, with studies typically finding only modest [2] to no change [7,9].

One reason could be that the manipulators tested do not allow their users to practice moving the limb in fully naturalistic ways, since they typically have a reduced number of degrees of freedom (DOF) compared to the human upper extremity, and functional tasks typically require coordinated motion of many joints in both the proximal and distal upper extremity [11]. Motor learning is also often task-specific [13], and training multiple functional tasks within one training session might be expected to yield better overall gains than training only one, less-functional task [4].

Exoskeletal robotic devices can be designed to follow the anatomy of the arm, and thus could assist in more naturalistic movements [11,14]. However, only a few studies to date have assessed the efficacy of exoskeletons on improving behavioral outcomes post-stroke. For example, studies and single-case studies of arm and hand exoskeletons found significant behavioral improvement that were maintained up to six months after completion of the exoskeletal training and that translated into a significant positive impact on activities of daily living [11,12]. Studies from our group that compared table-top exercise groups to both non-robotic (T-WREX, now ArmeoSpring [15]) and robotic (Pneu-WREX, [10]) three DOF exoskeleton-based training groups obtained significant functional gains of the trained upper limb after 8 weeks, and the gains obtained in the exoskeleton training groups tended to exceed those due to table-top exercises groups.

In the present study we examined the therapeutic effects of an exoskeleton that simultaneously trains the shoulder, elbow, forearm, wrist and hand. The goals of this study were twofold. First, we wanted to evaluate the effect of a training program that used the exoskeleton to train both the proximal and distal arm on behavioral outcomes and its impact on activities of daily living post-stroke. We hypothesized that the robotic exoskeletal training would be beneficial for increasing the function of the trained limb, having a positive impact on ADLs, and that those gains would be maintained over time. Secondly, we wanted to evaluate the relative impact of two different types of robotic training —multijoint functional and single joint training—on the ability to move the arm. We hypothesized that multijoint functional robotic training that included a variety of computer games imitating different activities of daily living would have a greater impact on the overall gains of the trained limb than training focused solely on single joint robotic training, implemented as tracking of a virtual phantom of individual joints, one at a time.

## Methods

### Inclusion/exclusion criteria

To be included in this study, subjects had to meet the following entry criteria: 1) age 18–73 years; 2) history of a unilateral stroke at least three months prior; 3) be able to lift, move and drop at least two blocks on the Box and Block Test over 60 seconds. Exclusion criteria were 1) contracture of the upper extremity (modified Ashworth scale >4); 2) significant subluxation or pain in the affected shoulder (score < 1 on the pain section of the Fugl-Meyer); 3) inability to passively abduct or flex the affected shoulder to 90 degrees without pain; 4) severe neglect [16], apraxia [17], and severe sensation deficit (score of 25/34 on the Nottingham Sensory Assessment) [18] sufficient to preclude repetitive reaching to visual targets; 5) any substantial decrease in alertness, language reception, or attention (score ≥ 1 (question 1) or score of 3 (question 9) on the NIH Stroke scale) [19]; 6) concurrent severe medical problems (including neurological, cardiovascular, orthopaedic, or psychiatric problems); 7) anti-spasticity medication changes six weeks prior to or during study; 8) current participation in other rehabilitation therapy. Informed consent was obtained from each subject before the evaluation session, and the University of California at Irvine Institutional Review Board approved the study.

### Randomization and robotic training program

**Figure 1:**
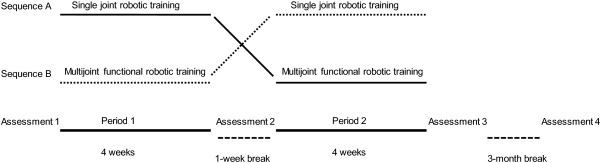
**Crossover study design.** All participants took part in the single joint and multijoint functional robotic training programs, with the order randomized. Subjects in Sequence A received single joint robotic training first, followed by a 1-week break, and then received multijoint functional robotic training. Subjects in Sequence B participated in the multijoint functional robotic training first, followed by a 1-week break and then participated in the single joint robotic training. Each period of training lasted 4 weeks. Two clinical assessments were conducted at baseline (Assessment 1), one after the first robotic training period (Assessment 2), one after the second robotic training period (Assessment 3) and one at a 3-month follow-up assessment (Assessment 4).

Subjects were assessed at baseline using the Fugl-Meyer Arm Motor Scale (FMA). They were stratified into three blocks based on their FMA score (30–40, 41–50, 51–66), and then, within each block, subjects were randomized to receive either multijoint functional robotic training or single joint robotic training first, using a randomization table generated by a blinded statistician. Following enrollment, training was conducted 3X/week for 60 minutes per session (an amount consistent with American College of Sports Medicine and American Heart Association guidelines [20]) for eight weeks for a total of 24 sessions. After four weeks of training, and a one week break, the subjects switched to the other training program. A trained physical therapist supervised each hour long training session (see Figure 1).

Both multijoint functional and single joint robotic training programs used the Biomimetic Orthosis for the Neurorehabilitation of the Elbow and Shoulder (BONES), a pneumatically-powered arm exoskeleton that allows movement at the shoulder (flexion/extension, horizontal abduction/adduction, external/internal rotation), and elbow (flexion/extension), allowing the arm to move through normal ranges of motion [21]. It does not use a ring bearing for shoulder internal/external rotation, but rather uses a parallel mechanism with mechanically grounded actuators to allow shoulder joint rotation. This mechanism design allows the device to have a low apparent inertia, since the shoulder actuators are mechanically grounded, and to generate large joint torques at the shoulder, since multiple actuators act together via the parallel mechanism. A pneumatic forearm/wrist exoskeleton – the Supinator Extender [22] – was attached to BONES to measure and assist in forearm pronation/supination and wrist flexion/extension. A pressure sensitive gripper was also connected to BONES to allow detection of hand grasp and release. For this study, BONES was controlled with an assistance-as-needed algorithm developed previously [23]. This algorithm forms a computer model in real-time of the amount of assistance the subject needs to complete a task, and prevents slacking by the subject by including a forgetting term for the model.

**Figure 2:**
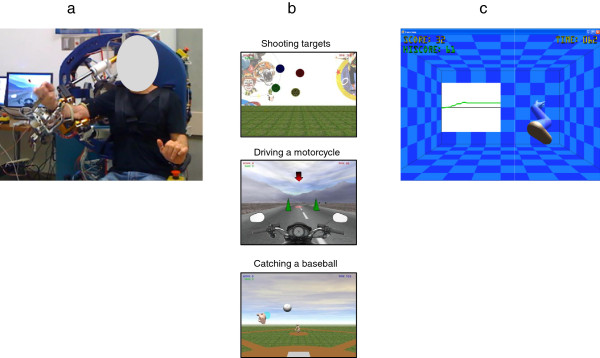
**BONES exoskeleton and examples of each robotic training program. a)** Subject training on BONES (Written informed consent was obtained from the subject for the publication of his picture); **b)** examples of games played during multijoint functional robotic training, **c)** example of single joint robotic training (shoulder flexion/extension).

The single joint robotic training consisted of tracking a 3D upper limb phantom shown on a computer screen, one joint DOF at a time (Figure 2). The training was grouped into blocks of 6 movements. One block consisted of 10 repetitions of each movement at each of the four joints (shoulder, elbow, forearm and wrist—one at a time), repeated 6 times over the 60-minute training session. The subject’s score was displayed on the computer screen after each trial and increased with the subject’s accuracy in tracking the phantom. The speed and range of motion of the phantom were fixed and identical for all subjects. Hand grasp was not exercised during single joint training.

The multijoint functional robotic training was comprised of 40 minutes of computerized games simulating functional activities and 20 minutes of the single joint robotic training. The functional games consisted of catching a baseball, driving a motor cycle, shooting targets, dropping marbles down a pegged board, playing air hockey, making an omelet, dunking a basketball, and tracking a moving target (pursuit rotor game). The games required the coordination of multiple joints of the affected upper limb, including hand grasp (see Figure 2). The trained physical therapist increased the level of difficulty of each game as appropriate, except the baseball and pursuit rotor games, for which software controlled the level of difficulty by altering the required speed of movements of the subject.

### Outcome measures

Subjects were assessed using standard clinical assessments by a trained, blinded therapist, and using quantitative measures of strength and speed of reaching. Two baseline assessments were taken before starting training; since we found a stable baseline (see below) we retained only the first of these assessments, and named it “Assessment 1” (see Figure 1). Another assessment was taken after four weeks of the first type of robotic training (Assessment 2), after the subsequent four weeks of the other type of robotic training (Assessment 3), and at a 3 month follow-up taken after completion of the second robotic training (Assessment 4). Additional robot-based assessments were performed at the beginning of each training week.

The clinical assessments were the Box and Block Test (BBT) [24], the FMA and Joint Pain Scale [25], the Wolf Motor Function Test (WMFT) [26], the Motor Activity Log (MAL) [27], grip and pinch strength, an assessment of apraxia [17], the Line Cancellation Test [16], the NIH Stroke Scale [19], the Nottingham Sensory Assessment [18], the modified Ashworth Scale [28], and the 10-m Walk Test [29]. The BBT and the FMA were assessed again on average three days after the first assessment to confirm motor function stability.

For the robot-based evaluations, subjects performed a strength test within the BONES exoskeleton for which they had to produce maximal voluntary concentric contractions at the shoulder (horizontal abduction/adduction, flexion/extension, internal/external rotation), elbow (flexion/extension), forearm (pronation/supination), and wrist (flexion/extension) by moving their affected upper extremity in the desired direction. The subjects’ joint torque production was shown to them by means of the length of a moving green rectangle. Similar to the protocol developed in Dewald et al. [30] to prevent use of abnormal synergies to generate torque, any compensation by other muscle groups (e.g., shoulder abductors while performing shoulder flexion) was not allowed and shown to the subjects by moving red rectangles that indicated joint movement in the undesired directions. These rectangles had to be within a window that represented ±5° from the desired joint position for the joint torque to be considered uncompensated. For each testing condition, the highest uncompensated joint torque of three trials was retained.

Subjects also performed a computerized reaching task in the BONES exoskeleton, or speed test, in which they reached for items located on a virtual shelf, shown at random locations. Once an item was grabbed using the virtual hand, the subjects had to move it, as fast as possible, into a shopping cart. Subjects performed the test once. The total time required to reach all objects was recorded. During this test, BONES cancelled its own weight but did not assist the subjects in moving their arms.

The primary outcome measure of the study was the change in BBT score, measured as the change in the number of blocks moved by the subject over 60 seconds. Secondary endpoints were the changes in the FMA, the WMFT, the MAL, the modified Ashworth Scale, the grip and pinch strength, and the robot-based strength and speed tests.

At the end of the study, subjects also filled out a survey, recording, on a 0 (not at all) to 5 (totally) Likert scale, their overall appreciation of the robotic training, its impact on the motion of their affected upper limb, and its influence on the performance of their daily activities. They were also asked to indicate whether they preferred the single joint robotic training, the functional robotic training or both.

### Statistical analysis

The subjects enrolled in this study were all at least three months post-stroke, and it was expected that they would have a stable baseline of motor ability. We confirmed this stability by comparing the two baseline scores for the BBT and FMA using a paired t-test. Normality of data was assessed by the Kolmogorov-Smirnov test. If any non-normal data was detected that could not be log transformed, appropriate non-parametric testing was used (Friedman’s ANOVA, Wilcoxon rank-sum and signed-rank tests). To assess the overall impact of BONES robotic training on behavioral improvement, a repeated measure ANOVA, with factor time (Assessment 1, Assessment 3, and Assessment 4) was done. For any significant ANOVA (p ≤ 0.05), a planned contrast with adjusted probability values (Bonferroni adjustment) was performed to locate the difference. To assess the effect of type of robotic training on the behavioral outcomes, three statistical approaches were used, ranging from most conservative to most liberal. First, a two-sample t test was used to compare the difference in the change in score for the first period of training (Assessment 2 – Assessment 1) between both groups. This analysis essentially ignored the crossover design, and simply looked at which training type was better during the first training period alone using the standard between-subjects approach. Second, the change in score due to each training period (change in score in period 1 = Assessment 2- Assessment 1; change in score in period 2 = Assessment 3 - Assessment 2) was calculated and the Hills-Armitage approach to crossover study analysis was used [30] to assess the effect of type of training on the behavioral outcomes. Thus, a two-sample t test was used to compare the change in score between the two sequences of training (AB vs. BA). The inequality of carryover effect was also assessed by comparing the sum of the values of the second and third assessments of robotic training between the two sequences. If any significant difference in the carryover effect was found between the two sequences, the first statistical approach described above (between group comparison on the change in score for the first period of training) was used as a reference to confirm the absence of difference between the two types of training. Third, we made the assumption that a carryover effect from the second to the third assessment did exist but was constant; that is, the gains experienced in the first training period established a new stable baseline movement ability at the beginning of the second training period. Then, a one sample t-test (equivalent to a paired t-test) was performed to locate any difference between the change in score due to the multijoint functional robotic training and the change in score due to the single joint training. Depending on each subject’s order of training, change in score due to multijoint functional robotic training was determined as:

[(Multijoint functional training first: Assess 2- Assess 1) + (Single joint training first: Assess 3 - Assess 2)]

Change in score due to single joint robotic training was determined as:

[(Single joint training first: Assess 2 – Assess 1) + (Multijoint functional training first: Assess 3 - Assess 2)]

Principle component analysis (PCA) was used as a further measure of improvement for both the clinical and robotic assessments, as a means to summarize the changes in the clinical and robotic assessments, many of which are correlated with each other, as a single number. The basic concept of PCA is to represent a set of scalar outcome measures for each subject as a vector, then to identify the vector direction (i.e. the linear combination of outcome measures) across subjects that exhibited the most change (most variance). The PCA score is then the projection of each subject’s vector onto this direction. The primary and all of the secondary outcomes of the behavioral analysis were in used in PCA for the clinical measures. To find the principle component, data from the 4 assessments (baseline, mid-training, post-training, and follow-up) of each outcome measure and all subjects were used. The data were first whitened for use in the PCA—meaning the *z*-score of each data point relative to other data points for an outcome measure was computed. Finally, each subject’s score along the principle component was converted to a *z*-score along that direction, so the units are the standard deviations of change in that direction. A total of 12 measures of robotic measurement of strength (flexion and extension of six joints), termed the coordinated measure of strength (CMS), were used for the PCA of robot outcome measures of strength. Here 8 assessments of each outcome measure for all subjects were used. Again, the data were whitened, and the *z*-scores along the principle component were found. Statistical analysis was performed using SPSS 18®.

## Results

Twenty subjects with chronic stroke (range: 8–156 months post stroke) met the inclusion criteria (Table 1). The repeated baseline measurements of the BBT and FMA were stable over time (p > 0.05). Therefore, the first baseline measurement was retained for analysis. No significant difference was noted for the baseline demographic and clinical scores between the two groups, except for the BBT, grip strength and strength of the shoulder abductors, in favor of the group that received single joint robotic training first (see Table 2). Note that when the baseline BBT scores were calculated as a percentage of the score for the unaffected arm, the difference between groups was not significant (p = 0.12).

**Table 1:** Demographic characteristics and assessments’ scores (mean ± SD) at baseline (Assess 1), at the end of the 8-week robotic training (Assess 3) and at 3-month follow-up (Assess 4)

**Demographic characteristics**	**All (n = 20)**				
Age (years)	60 ± 7				
Time since stroke (months)	38 ± 38				
Gender (Male [M]/ Female [F])	12 M/8 F				
Side of hemiparesis (Right/Left)	14/6				
NIH Stroke Scale Score (normal = 0)	3 ± 2				
Line Cancellation Test (normal = 0)	0 ± 0				
Nottingham Sensory Assessment (max = 34)	33 ± 3				
10-meter Walk Test (m/s)	1.3 ± 0.5				
Assessments	Assess 1	Assess 3	Assess 4	p*	p**
Box and Block Test (# blocks in 60 s)	31 ± 13	37 ±13	36 ± 12	p < 0.05	p = 0.6
Fugl-Meyer Arm Motor Scale (normal = 66)	52 ± 8	55 ± 7	55 ± 7	p = 0.01	p = 0.2
Wolf Motor Function Test					
Score (max = 5)	3.9 ± 0.6	4.3 ± 0.5	4.3 ± 0.5	p < 0.05	p = 1.0
Time to completion (max = 1800 s)	7.9 ± 11.6	4.5 ± 5.8	4.5 ± 6.7	p = 0.001	p = 0.5
Weight (max = 20 lbs)	13 ± 6	15 ± 5	15 ± 5	p = 0.001	p = 0.5
Motor Activity Log					
Amount of use (max = 5)	2.7 ± 1.0	3.3 ± 1.1	3.5 ± 1.2	p = 0.001	p = 0.1
Quality of movement (max = 5)	2.4 ± 1.1	3.1 ± 1.1	3.1 ± 1.1	p < 0.05	p = 1.0
Grip strength (kg)	18.8 ± 14.0	20.6 ± 14.0	22.0 ± 14.9	p = 0.008	p = 0.1
Pinch strength (kg)	4.3 ± 2.5	4.5 ± 2.4	4.3 ± 2.3	p = 0.008	p = 1.0
Modified Ashworth Scale					
Shoulder (normal = 0)	0.4 ± 0.7	0.2 ± 0.7	0.1 ± 0.4	p = 0.05	p = 0.3
Elbow (normal = 0)	0.8 ± 1.0	0.7 ± 0.9	0.4 ± 0.8	p = 0.4	p = 0.03
Time of execution of a robotic reaching task (s)	75.0 ± 40.9	53.2 ± 29.5	n/a	p < 0.05	n/a
Maximal voluntary concentric strength (Nm)					
Shoulder abductors	29 ± 14	30 ± 12	n/a	p = 0.8	n/a
Shoulder adductors	23 ± 11	32 ± 10		p < 0.05	
Shoulder internal rotators	12 ± 8	16 ± 8		p = 0.02	
Shoulder external rotators	5 ± 4	10 ± 7		p < 0.05	
Shoulder flexors	25 ± 10	27 ± 13		p = 0.3	
Shoulder extensors	38 ± 11	43 ± 9		p = 0.06	
Elbow flexors	23 ± 8	29 ± 10		p = 0.001	
Elbow extensors	19 ± 11	23 ± 12		p = 0.06	
Forearm supinators	3 ± 2	4 ± 3		p = 0.03	
Forearm pronators	8 ± 5	9 ± 5		p = 0.2	
Wrist flexors	6 ± 4	6 ± 4		p = 0.3	
Wrist extensors	6 ± 4	7 ± 4		p = 0.03	

*p value between baseline (Assess 1) and 8 weeks of robotic training (Assess 3); ** p value between 8 weeks of robotic training (Assess 3) and 3-month follow-up (Assess 4).

**Table 2:** Assessment measurements taken at baseline (Assess 1), after 4 weeks (Assess 2) and 8 weeks (Assess 3) of robotic training for each group

	**Single joint robotic training first group (n = 10)**			**Multijoint functional robotic training first group (n = 10)**			**p values**			
Assessment	Assess 1	Assess 2	Assess 3	Assess 1	Assess 2	Assess 3	p^a^	p^b^	p^c^	p^d^
Box and Block Test (# blocks in 60 s)	36 ±14	42 ±14	43 ±13	25 ± 11	29 ± 8	31 ±11	0.05	0.5	0.6	0.4
Fugl-Meyer Arm Motor Scale (normal = 66)	52 ± 9	53 ± 8	55 ±8	52 ± 6	54 ± 6	56 ±7	1.0	0.3	0.8	0.5
Wolf Motor Function Test										
Score (max = 5)	4.1 ± 0.7	4.4 ± 0.6	4.5 ± 0.5	3.7 ± 0.5	4.0 ± 0.4	4.1 ± 0.4	0.1	0.7	0.6	0.6
Time to completion (max = 1800 s)	7.9 ± 14.6	5.8 ± 11.5	4.3 ± 7.8	7.8 ± 8.4	5.7 ± 5.1	4.6 ± 3.2	1.0	0.3	0.1	0.3
Weight (max = 20 lbs)	14 ± 7	15 ± 6	16 ± 5	12 ± 5	14 ± 5	15 ± 4	0.4	0.2	0.7	0.5
Motor Activity Log										
Amount of use (max = 5)	2.9 ± 1.2	3.7 ± 1.2	3.6 ± 1.2	2.6 ± 0.9	3.0 ± 0.9	3.1 ± 1.0	0.5	0.4	0.4	0.5
Quality of movement (max = 5)	2.7 ± 1.2	3.5 ± 1.1	3.5 ± 1.2	2.1 ± 0.8	2.4 ± 0.7	2.7 ± 0.9	0.2	0.2	0.2	0.5
Grip strength (kg)	25.6 ± 16.3	27.1 ± 14.0	27.6 ± 16.2	11.9 ± 6.7	13.1 ± 7.2	13.6 ± 6.7	0.02	0.2	0.9	0.9
Pinch strength (kg)	5.2 ± 2.6	6.0 ± 2.7	5.7 ± 2.5	3.5 ± 2.1	3.6 ± 1.9	3.6 ± 1.8	0.1	0.001	0.001	0.02
Time of execution of a speed task (s)	77.5 ± 54.6	60.9 ± 43.5	56.4 ± 40.1	72.6 ± 23.3	60.7 ± 22.8	50.1 ± 14.2	0.4	0.5	0.8	0.9
Maximal concentric strength (Nm)										
Shoulder abductors	36 ± 14	35 ± 12	34 ± 12	22 ± 9	26 ± 12	25 ± 11	0.02	0.4	0.6	0.9
Shoulder adductors	26 ± 12	30 ± 10	35 ± 9	20 ± 8	26 ± 9	30 ± 11	0.2	0.5	0.4	0.4
Shoulder internal rotators	15 ± 9	18 ± 7	20 ± 8	10 ± 3	11 ± 3	13 ± 6	0.1	0.5	0.5	0.5
Shoulder external rotators	6 ± 4	11 ± 7	12 ± 8	4 ± 3	7 ± 6	7 ± 6	0.2	0.4	0.9	0.9
Shoulder flexors	28 ± 10	30 ± 13	32 ± 15	21 ± 8	24 ± 9	23 ± 10	0.1	0.7	0.4	0.4
Shoulder extensors	40 ± 12	44 ± 10	45 ± 10	36 ± 10	36 ± 10	41 ± 8	0.5	0.5	0.2	0.1
Elbow flexors	27 ± 9	29 ± 10	31 ± 11	19 ± 4	23 ± 8	27 ± 8	0.07	0.5	1.0	1.0
Elbow extensors	23 ± 11	24 ± 10	26 ± 12	14 ± 9	18 ± 8	20 ± 11	0.06	0.4	0.5	0.5
Forearm supinators	3 ± 2	4 ± 3	4 ± 3	3 ± 2	3 ± 1	4 ± 2	0.7	0.1	0.1	0.2
Forearm pronators	9 ± 5	9 ± 5	10 ± 5	7 ± 4	8 ± 4	7 ± 4	0.2	0.7	0.8	0.8
Wrist flexors	7 ± 4	7 ± 4	8 ± 4	5 ± 3	6 ± 4	5 ± 3	0.2	0.08	0.08	0.07
Wrist extensors	7 ± 4	8 ± 4	9 ± 4	4 ± 3	5 ± 3	5 ± 4	0.1	0.5	0.8	0.9

p^a^ = between groups comparison on the baseline scores (Assess 1).

p^b^ = between groups comparison on the difference in the change in score: Assess 2 – Assess 1.

p^c^ = between groups comparison on the difference in the change in score between the two sequences of training: AB sequence – BA sequence = 0.

p^d^ = within subjects comparison on the difference in the change in score: Multijoint functional robotic training – Single joint robotic training = 0.

### Behavioral improvement following the BONES robotic training

**Figure 3:**
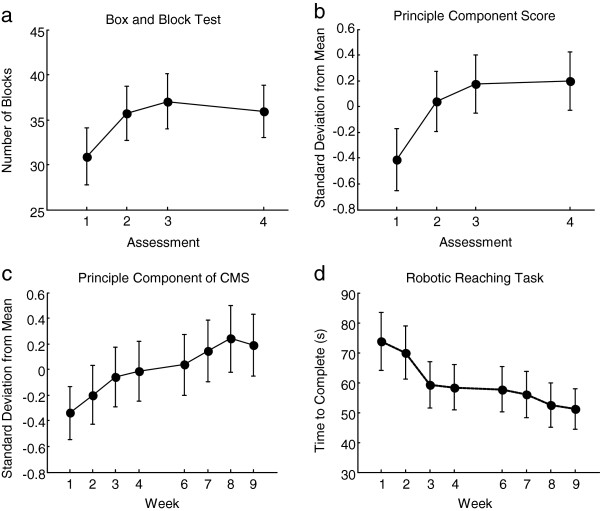
**Summary of clinical and robotic outcome measures as a function of time across all subjects. a)** The primary outcome measure of the study, the Box and Block Test. **b)** The principle component score of all clinical outcome measures over the duration of the study. **c)** The principle component scores for all maximum coordinated movement strength (CMS) measurements taken with the robot. **d)** The results for the robotic reaching task. Error bars represent standard error of the mean. The empty space at week 5 indicates the 1-week break. Assessments 1, 2, 3 and 4 correspond to assessments taken at baseline, after the first 4 weeks of training, after completion of 8 weeks of training, and at a 3-month follow-up, respectively.

Overall, when looking at the difference in number of blocks carried at the BBT between Assessment 3 and Assessment 1, a significant mean improvement in the BBT score was noted (gain of 6 blocks, p<0.05), that was maintained at Assessment 4 (see Table 1 and Figure 3). Regarding the secondary outcome measures, they all significantly improved after the BONES robotic training, and all the gains were maintained at Assessment 4. Only spasticity at the affected elbow did not change from Assessment 1 to Assessment 3, but did improve at Assessment 4. The single score summarizing all outcome measures obtained via PCA also showed improvement with training and maintenance at the Assessment 4 (Figure 3).

For the robotic assessment, a significant decrease in the time of completion of the reaching task was found after completion of the BONES robotic training (p<0.05). For the robotic strength assessment, a significant increase in maximal voluntary concentric strength was noted for the shoulder adductors (p < 0.05), internal (p = 0.02) and external (p<0.05) rotators, elbow flexors (p = 0.001), forearm supinators (p = 0.03) and wrist extensors (p = 0.03). Again, the single score summarizing all strength outcome measures obtained via PCA showed improvement across the training period (Figure 3).

### Impact of the type of robotic training on behavioral outcomes

**Figure 4:**
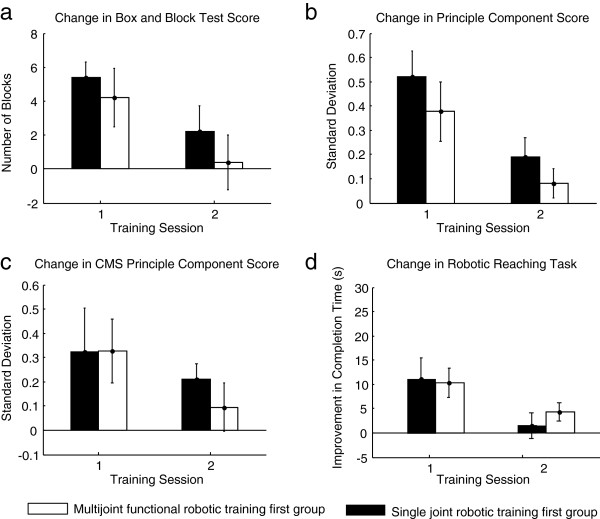
**Results of each training type for the clinical and robotic measurements. a)** The primary outcome measure of the study, the Box and Block Test. **b)** The principle component score of all clinical outcome measures over the duration of the study. **c)** The principle component scores for all coordinated movement strength (CMS) measurements. **d)** The time of completion of the robotic reaching task. Error bars represent standard error of the mean.

Regardless of the statistical approaches used, results showed that the multijoint functional robotic training was not superior to the single joint robotic training for improving the BBT score. The same results were noted for all the secondary outcome measures, except for the pinch strength, in favor of the single joint robotic training (see Table 2). For the robotic assessments, no significant difference between the two robotic training programs was noted for the decrease in the time of completion of the unassisted reaching tasks as well as the strength gains at the upper limb (see Table 2). The PCA-based summary measurement of all clinical and robotic measurements showed no difference with training type either (Figure 4).

When looking at the differences in carryover effect between the two sequences (AB vs. BA) using the Hill-Armitage approach, results revealed significant inequality of carryover effects for the BBT (p = 0.02), the WMFT time to completion (p = 0.01), the MAL “Quality of movement” subsection (p = 0.02), the grip (p = 0.02) and pinch (p = 0.04) strength tests, and the maximal voluntary concentric strength at the shoulder internal rotators (p = 0.01) only. For all these outcome measures, the AB sequence (single joint robotic training first) showed greater carryover effect than the BA sequence (multijoint functional robotic training first). However, when looking at the independent t-tests performed on the change in score for the first period of training (Assessment 2 – Assessment 1), no difference between multijoint functional robotic training and single joint robotic training was noted for all these outcome measures, except for pinch strength, where a significant difference between the two robotic training programs was found (0.2 ±0.4 vs. 0.8 ±0.4; p = 0.001) in favor of the single joint robotic training.

### Post-therapy survey outcome

Two subjects did not complete the survey for a total of 18 respondents. When asked if they enjoyed training on the robot, all subjects rated 5/5. When asked if they noticed any improvement in the motion of their affected limb, 44% of them rated 4/5 and 38% 5/5. More subjects rated 5/5 (41%) when asked if the gains obtained after the robotic training translated to improvement of daily activities. Two examples of improvements mentioned by the subjects: ‘My left [affected] arm can now help me to wipe and wash my face’; ‘Before BONES I could barely use my hand [affected] but now I can flip pancakes for my daughters and grandsons’. Finally, 6% of the subjects wrote that they preferred the single joint robotic training over multijoint functional robotic training, 19% multijoint functional robotic training over single joint robotic training and 75% rated both trainings equally.

## Discussion

This study evaluated the impact of BONES robotic training on behavioral outcomes of the affected upper limb after stroke, and assessed if multijoint functional robotic training was more effective in improving behavioral performance than single joint robotic training. The results indicated that the overall program of BONES training was effective in improving manual dexterity at the affected upper limb, and that the gains were maintained after 3 months of completion of the study (Assessment 4). Improvements in the secondary outcome measures were also noted, and maintained over time. In addition, BONES robotic training had a significant and positive impact on the use and quality of movement of the trained upper limb as self-reported by subjects during their ADL performance. When comparing the impact of the type of robotic training on behavioral gains, results showed no difference between the multijoint functional robotic training and the single joint robotic training. All together, these findings suggest that robotic training of the whole affected upper limb after a stroke can improve behavioral outcomes and subjects’ self-perceived ADL performance, but the specific importance of multijoint functional training versus individualized joint training is less clear.

### Robotic therapy and motor function

This study further supports the use of high-intensity and repetitive robotic exoskeletal training not only to reduce motor impairment but also to enhance motor function post-stroke [11]. All subjects but one in the current cohort improved at the BBT. Looking further at the baseline characteristics of this subject, he had the least difference in the number of blocks carried between the affected and unaffected upper extremity (affected/unaffected side: 50/55 blocks; data not presented). He also had the highest baseline FMA score (63/66), and WMFT score (4.9/5). In Staubli et al. [11], the most impaired subject also did not gain much with the robotic training, and thus it could be hypothesized that robotic training is effective within a certain range of levels of recovery, where mild and severely impaired individuals might not benefit as much. A companion study is being done to establish which baseline variables were predictive of positive final behavioral gains from robot intervention in order to help maximise individual selection for such therapy.

Along with positive change in function as measured with the BBT, the robotic training allowed significant reduction in motor impairment, as evaluated mainly by the FMA assessment, further supporting results of previous studies (e.g. [10-12,15]). However, the improvement in motor impairment was more modest in our study than in others. Indeed, we reported a mean 3-point gain in the FMA score as opposed to gains of up to 17.6 points [11,12]. This discrepancy could be explained by the fact that the current subjects had mild to moderate baseline motor impairment at the affected upper extremity as measured by this scale (mean FMA: 52/66), leaving a smaller window of improvement and a potential greater impact of the ceiling effect of the FMA [25].

Although improvement in motor impairment, as assessed in a research setting, does not automatically imply a greater use of the affected arm in everyday life [31], BONES training is one of the few robotic training programs that had a positive impact on the subjects’ performance in ADL, as 17 out of 20 subjects stated, on the MAL, that they were able to use their trained limb more and better in daily tasks. Because BONES allows the arm to move through the normal wide workspace, as opposed to some end-effector robots [2] or other limited DOF exoskeletons [10], it could be thought that training the upper limb in shoulder, elbow, forearm, wrist, and hand DOF increases the chances of a transfer of gains to ADL. Another reason could be that the significant gains in strength in muscle groups playing an important role in the upper-limb function [32], such as the shoulder adductors and elbow flexors, combined with a significant improvement in the speed of execution of a reaching task, might have facilitated the carry-over benefits to ADL. Despite being in the chronic phase of their stroke, participants benefited from the robotic intervention in their daily life, challenging once again the idea that no further improvement can be induced after spontaneous recovery reaches a plateau, such as at 6 months post-stroke [33]. It seems that providing new, challenging, intense and repetitive training can trigger further functional recovery in this population [33].

### Multijoint functional versus single joint robotic training

Conventional clinical wisdom is that rehabilitation should progress from simpler movements to more complex, functional ones as recovery progresses. If so, functional movements should have been a more effective training paradigm for higher level subjects, such as the population of subjects enrolled in the current study, but this was not the case. Moreover, it is thought that subjects will retain more from training when asked to perform various tasks within one training session rather than training one single task. The reasoning is that task variability triggers the brain to ‘solve a problem in each trial rather than replaying a movement from memory’ [4]. Task variability within a training session is also known to promote generalization to other tasks [34]. Following this line of reasoning, multijoint functional robotic training should have been better than single joint robotic training because it forced practice of a variety of functional tasks that required many different types of movements, coordination of multiple joints and muscle groups, and various “problems to be solved”, whereas single joint robotic training simply required following a phantom joint in a stereotypical movement over and over.

The sample size of this pilot study was limited, but, with this caveat, we speculate that perhaps task-specificity is not the sole key factor in promoting positive gains after stroke. We briefly discuss several other recent studies that support this viewpoint. First, in a recent study comparing the transfer of training of a functional task (feeding) to untrained spatiotemporal similar (sorting) or different (dressing) tasks, significant improvement in all the three tasks was noted after training exclusively on one functional task. The authors suggest that matching the characteristics of a task during training to the target tasks to be improved is not important to promote functional gains and transfer after stroke [35]. Second, in a study comparing impairment-based robotic training to functional robotic training in chronic stroke survivors, similar significant reduction in motor impairments of the affected upper limb was found with both types of training [8]. These researchers speculated that functional robotic training might require a higher degree of attention from subjects than single joint robotic training and this might be too challenging for some subjects, preventing greater benefit from functional training.

As a third example of how other factors besides task specificity are important, another study failed to show any significant difference in improvement of reaching ability between robot-assisted reaching (reaching along a linear guide) and free reaching groups (i.e. task specific practice) in chronic stroke survivors. The authors suggested that the action of trying to move the affected limb might be a more fundamental stimulus of movement recovery rather than the type of training provided [7]. This concept is also supported by our recent finding that repetitive practice of a highly stereotypical arm movement facilitated by a lever-like mechanical device attached to a manual wheelchair can have substantial benefits for individuals with chronic stroke [36]. The same idea is supported as well by a recent study on task-specific training of the lower-limb muscles in chronic stroke survivors where similar gains in gait performance were obtained between the lower-limb training group and a control group training the upper-limb muscles [37].

We also recently conducted an experiment in which unimpaired subjects tried to learn a novel multi-joint movement with the BONES exoskeleton [38]. We found better learning of the movement when the task was decomposed in simpler parts as opposed to practicing only the whole; that is, practicing only the whole multi-joint task was less effective than a repetition-matched amount of practice that included individual joint movement practice. In this case, then, as can also be observed in practical approaches to music and sports training, breaking down a movement was better than a sole focus on practicing the task to be learned.

Caution then seems to be warranted in becoming overly focused on the task-specific training approach. That is, the above studies suggest that factors such as attention capacity, plasticity driven by effort alone, and amenability of motor learning processes to simplified forms of practice are also important and may interact in a complex way to influence the relative effectiveness of task-specific training. Even at the level of subjective preference for training technique, we were surprised that subjects in the present study typically identified both multijoint functional and single joint robotic training as equally valuable to them.

What then do the study results suggest for the desirability of complex multi-joint exoskeletons for robotic rehabilitation? Our original motivation for building BONES was to allow training of more functional movements. While we did not find a distinct advantage to more functional training, it may have been that exercising a large number of joints with BONES, albeit one at a time, was beneficial for promoting the significant functional gains we observed. It is possible that sophisticated, multi-joint exoskeletons will be useful because they make the exercise of more DOFs convenient, allowing a greater transfer of gains to improvement in ADL [11]. On the other hand, incorporating complex, task-specific, virtual games with these exoskeletons might not be essential to elicit improvement in function.

### Study limitations

This crossover study used a relatively small number of subjects, which made balancing groups challenging, especially for our main outcome measure. Even though an inequality of carryover effect was noted between groups for the BBT, the subsequent analysis on the change in score after the first period of training did not show any significant difference between the two types of robotic training. A study using a greater sample size and a longer washout period is needed to further support our current findings. Also, our subjects were, for the most, mildly to moderately impaired, although, on average, they moved about half the number of blocks in the BBT as a non-impaired subject, indicating still significant impairment. Even though the robotic training had a positive impact for these subjects, it would be desirable to assess the impact of such training for more impaired individuals who might need similar high-intensity training of their affected arm.

## Conclusion

Robotic training using a sophisticated exoskeleton improved behavioral outcomes of the affected upper extremity in chronic stroke survivors. Multijoint functional robotic training led to significant gains similar to single joint robotic training, challenging the need for robotic devices to incorporate virtual functional games, at least for chronic, moderately-to-mildly impaired subjects. Future studies might explore the effectiveness of training with the BONES exoskeleton for more impaired subjects as well as providing a randomized controlled trial comparing this robotic training with conventional therapy to determine if BONES training is more effective than conventional therapy.

## Competing interests

David Reinkensmeyer has a financial interest in Hocoma, A.G. and Flint Rehabilitaton Devices, companies that make rehabilitation devices. The terms of these arrangements have been reviewed and approved by the University of California, Irvine, in accordance with its conflict of interest policies. No other authors have any competing interests.

## Authors’ contributions

Conception and design the experiment: MHM, SJS, JPA, JK, JPB, SCC, DJR. Collection of the data: MHM, SJS, VC, CC. Analysis and interpretation of the data: MHM, SJS, SCC, DJR. Writing of the manuscript: MHM, SJS. Revising the manuscript: JPA, JK, JPB, SCC, DJR. All the authors read and approved the final manuscript.
